# Biomarker combination and SOFA score for the prediction of mortality in sepsis and septic shock: A prospective observational study according to the Sepsis-3 definitions

**DOI:** 10.1097/MD.0000000000020495

**Published:** 2020-05-29

**Authors:** Juhyun Song, Sungwoo Moon, Dae Won Park, Han-Jin Cho, Joo Yeong Kim, Jonghak Park, Jae Hyung Cha

**Affiliations:** a Department of Emergency Medicine, Korea University Ansan Hospital; b Division of Infectious Diseases, Department of Internal Medicine, Korea University Ansan Hospital, Ansan; c National Emergency Medical Center, National Medical Center, Seoul; d Medical Science Research Center, Korea University Ansan Hospital, Ansan, Republic of Korea.

**Keywords:** biomarker, combination, mortality, sepsis, septic shock, Sequential Organ Failure Assessment score

## Abstract

Biomarkers are valuable tools for the prediction of mortality in patients with sepsis. However, the use of a single biomarker to predict patient outcomes is challenging owing to the complexity and redundancy of the immune response to infections.

We aimed to conduct a prospective observational analysis to investigate the prognostic value of pentraxin 3, interleukin 6, procalcitonin, and lactate combined in predicting the 28-day mortality rate in patients with sepsis or septic shock (n = 160; sepsis, 78; sepsis shock, 82). Two methods (the frequency sum of values above the cutoff, and the multivariate logistic regression model) were used to assess the prognostic value of the biomarker combination.

In the receiver operating characteristic curve analyses, the combination of the 4 biomarkers was better than the Sequential Organ Failure Assessment (SOFA) score in predicting the 28-day mortality rate, regardless of whether the frequency sum of values above the cutoff or the multivariate logistic model was used for the analysis. The addition of the SOFA score to the biomarker combination did not result in a better performance for the prediction of mortality.

The combined biomarker approach showed good performance in predicting 28-day all-cause mortality among patients diagnosed with either sepsis or septic shock according to the Sepsis-3 definitions. Furthermore, it was superior to the SOFA score in predicting mortality.

## 1 Introduction

Sepsis is defined as a life-threatening organ dysfunction caused by a dysregulated host response to infection.^[1]^ Globally, it is a public health problem and remains the leading cause of death among critically ill patients, with an overall mortality rate of 30%.^[2,3]^ Currently, there is no gold standard to definitively diagnose sepsis; no tools to screen, evaluate, and optimize its treatment; and no reliable method to predict patient outcomes.^[4]^ Although the Sequential Organ Failure Assessment (SOFA) scoring system has been proposed for predicting mortality in patients with sepsis, it has been criticized for its limited ability to predict the patient outcomes.^[1,5]^ In a clinical setting, biomarkers can be of diagnostic and prognostic value and can aid in decision making about the appropriate treatment course to take. More than 100 biomarkers have been proposed to be useful for the detection of sepsis and the predication of mortality.^[6]^ Based on recent studies, four biomarkers (pentraxin 3 (PTX3), interleukin 6 (IL6), procalcitonin (PCT), and lactate) were selected from a large pool of potential biomarker candidates for the validation of their prognostic value. The selection criteria for the biomarkers are

1. previously validated biomarkers for predicting mortality in sepsis diagnosed at emergency department (ED) (all the markers),
2. an emerging biomarker for diagnosing sepsis (PTX3),
3. a biomarker acting as both a pro-inflammatory cytokine and an anti-inflammatory myokine (IL6),
4. a representative biomarker for bacterial infection (PCT), and
5. an established diagnostic marker for septic shock (lactate).

Serum lactate levels are known to be associated with 28-day mortality in patients with severe sepsis and septic shock.^[7,8]^ Recent meta-analyses have demonstrated that the baseline PCT levels and non-clearance were associated with mortality in patients with sepsis.^[9,10]^ PTX3, the representative member of the subfamily of long pentraxins, is an acute-phase protein and a novel biomarker.^[11]^ It is expressed in various cells (eg, endothelial cells, monocytes, dendritic cells, and neutrophils) during inflammatory processes.^[12]^ A recent study has shown that PTX3 has prognostic value for the prediction of mortality in patients with Sepsis-3-defined sepsis and septic shock.^[13]^ Furthermore, a previous study had shown that IL6 levels were associated with the extent of inflammation, severity of organ dysfunction, and sepsis-related death.

We aimed to conduct a prospective observational analysis to confirm the prognostic value of PTX3, PCT, IL6, and lactate in patients with sepsis or septic shock. However, the complexity and redundancy of the host immune response to infections make it challenging to assign risk profiles, stratify on the basis of pathophysiology, and predict outcomes with only a single biomarker.^[14,15]^ We hypothesized that a combination of biomarkers would be better than any single biomarker in predicting 28-day mortality. In particular, we investigated whether the addition of SOFA scores to the use of biomarkers could improve the prediction of mortality in patients with sepsis or septic shock.

## 2 Materials and methods

### 2.1 Study design and setting

A prospective observational study was conducted at the general ED of Korea University Ansan Hospital. The institution is an 820-bed, tertiary-care academic medical center with an annual census of approximately 50,000 patients.

### 2.2 Study population

**Figure 1:**
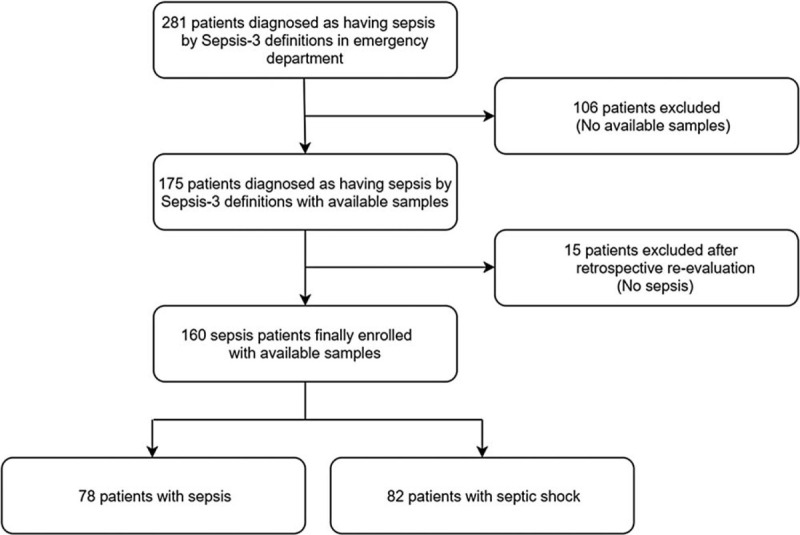
Flow chart of the process used for selecting the study participants.

From December 2017 to December 2018, 281 consecutive adult patients (≥18 years old) in the ED were clinically diagnosed with sepsis (as defined by Sepsis-3), using the Intelligent Sepsis Management System (i-SMS). The i-SMS is a quick SOFA (qSOFA) alert system that was developed to facilitate the early diagnosis of, and appropriate management for, sepsis in accordance with the latest Sepsis-3 definitions and 2016 Surviving Sepsis Campaign guidelines. During the initial study period, these 281 patients with clinically diagnosed sepsis met all of the following three conditions: initial positive qSOFA result, presence of infection, and a SOFA score ≥ 2. Among the 281 patients, 106 without available samples (refusal to participate in the study, 102; inappropriate samples, 4) were excluded from the study. The remaining 175 patients with available samples were recruited and their medical records, laboratory results, and radiologic findings were reviewed, whereupon 15 patients were excluded because they did not meet the diagnostic criteria for sepsis as defined by Sepsis-3. Therefore, 160 patients were finally enrolled in the study (Fig. 1). These individuals all received standard-of-care management in the emergency department according to the 2016 Surviving Sepsis Campaign guidelines.

The present study was approved by the Institutional Review Board of Korea University Ansan Hospital (IRB No. 2019AS0188) and conducted in accordance with the Declaration of Helsinki. A written informed consent was obtained from either the patients or their legal representatives before the recruitment.

### 2.3 Data collection and biomarker measurement

For each patient, the demographic information (age, sex, and prior medical history), vital signs, routine laboratory test results (creatinine, bilirubin, platelet count, C-reactive protein, hemoglobin, hematocrit, sodium, potassium, urea, white blood cell count, and blood culture), biomarker measurements (lactate, PCT, PTX3, and IL6 levels), blood gas analysis results (pH, PaO_2_, PaCO_2_, bicarbonate, and base excess), Glasgow Coma Scale score, and outcome (28-day mortality) were collected and documented. The SOFA score was calculated for each patient upon initial assessment in the ED.

The biomarker measurements were carried out using the residual blood samples from tests conducted during routine laboratory evaluation. All blood samples for the lactate, IL6, PTX3, and PCT measurements were obtained within 6 hours of the clinical diagnosis of sepsis. Whole blood was collected into serum-separating tubes, following which aliquots of the separated serum were frozen at −80°C until analysis. The samples were kept on ice before the measurement of the serum biomarker levels (in duplicates). The serum PCT levels were measured using commercial reagents (Thermo Fisher Scientific Clinical Diagnostics, BRAHMS GmbH, Hennigsdorf, Germany). The serum IL6 levels were measured using a commercially available enzyme-linked immunosorbent assay (ELISA) (R&D Systems, Minneapolis, MN), where the inter- and intra-assay coefficients of variability were 4.5 ± 1.7% and 2.6 ± 1.4%, respectively. The serum PTX3 levels were measured using a commercially available ELISA (R&D Systems, Minneapolis, MN), where the inter- and intra-assay coefficients of variability were 5.1 ± 1.1% and 3.9 ± 0.4%, respectively. The lactate level was measured with a serum-based assay catalyzed by lactate oxidase (Vitro, Ortho Clinical Diagnosis, Rochester, NY).

### 2.4 Definitions

Sepsis-3 definitions recommend the use of a qSOFA score for the screening of sepsis in patients outside of the intensive care unit. The qSOFA score, which ranges from 0 to 3 points, uses the following three criteria: assigning 1 point for low blood pressure (systolic blood pressure ≤100 mmHg); a high respiratory rate (≥22 breaths per minute); or an altered mental status (Glasgow Coma Scale < 15). A positive qSOFA score, implying the presence of 2 or more qSOFA points near the onset of infection, was included in the inclusion criteria in the present study. According to the Sepsis-3 definitions, the diagnostic criteria for sepsis include an increase in the SOFA score of 2 points or more as a result of the current infection. The criteria for septic shock include the need for a vasopressor to maintain a mean arterial pressure of 65 mmHg, and a serum lactate level of greater than 2 mmol/L, despite adequate fluid resuscitation. In accordance with the 2016 Surviving Sepsis Campaign guidelines, the serum lactate levels were measured in all the patients.

### 2.5 Statistical analysis

Based on the results of the previous study, we expected 28-day all-cause mortality to be 35%. The previous study showed that multi-marker approach predicted mortality better than SOFA score (area under the receiver operating characteristic curves [AUC], 0.769 vs 0.615).^[16]^ Our hypothesis was that we would observe similar AUCs in our study. Assuming 90% power with a 2-sided alpha levels of 0.05, our study required 147 patients (94 survivors and 53 non-survivors). Continuous variables were expressed as the median and interquartile range or the mean ± standard deviation according to their distributions. Groups of each biomarker were compared using the Mann–Whitney *U* test. The receiver operating characteristic (ROC) curves of the SOFA scores and of each biomarker were explored to explore the optimal cutoff values for the prediction of 28-day all-cause mortality. The optimal cutoff value indicates where the sum of sensitivity and specificity is the highest (Youden index). AUCs were expressed with their 95% confidence intervals (CIs). The SOFA score and each biomarker were dichotomized into above (1 point) and below (0 point) cutoff values according to the respective optimal cutoff values for 28-day mortality. Univariate and multivariate Cox proportional hazards models were constructed to derive risk factors for 28-day all-cause mortality, where the unadjusted and adjusted hazard ratios (HR, with 95% CI) of the dichotomized variables were obtained. The patients were divided into 6 groups according to the frequency sum (total points) of values above the cutoff, and each group was compared in terms of the 28-day mortality rate. Overall, the patients were divided into a high-score group and a low-score group. Kaplan–Meier curves of these 2 groups were generated, and the log-rank test was used to compare the 30-day mortality rates. Using dichotomized variables for the cutoff value for 30-day mortality for the respective biomarkers, ROC curves for the combined biomarkers, SOFA scores, and combination of biomarkers and SOFA scores were generated and their AUC values were compared to predict the 28-day mortality rate.

In addition, a logistic regression equation was constructed to predict the probability of 28-day mortality. For this, all biomarkers with a univariate significance of *P* < .10 as covariates and 28-day all-cause mortality as the dependent variable were included using the backward elimination method. The logistic regression equation for predicting a logit transformation (Logit(p)) of the probability of 28-day mortality was created using the coefficients generated for each biomarker in the final step of the regression model. Finally, the Logit(p) value was converted to the probability of 28-day mortality using the following conversion method: 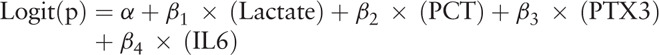


The Hosmer–Lemeshow goodness-of-fit test was used to evaluate the fidelity of the model. The AUC and CI of the probability of 28-day mortality were calculated. The study participants were divided into 2 groups according to the optimal cutoff value of the probability of 28-day mortality, and then the 28-day mortality rates of the groups were compared using Kaplan–Meier curve analysis. A log-rank test was conducted to compare the survival curves of the groups.

Subgroup analysis was conducted according to the disease severity (sepsis vs septic shock). Two sensitivity analyses were conducted to test the model assumptions. First, we conducted a sensitivity analysis using the Sepsis-2 definitions and included severe sepsis and septic shock in accordance with the previous definitions. Second, we conducted a sensitivity analysis excluding patients who were transferred from other hospitals.

All analyses in this study were performed using the MedCalc for Windows, version 19.1.6 (MedCalc Software, Mariakerke, Belgium) and SPSS version 23.0 (IBM, Armonk, NY). A *P* value of less than .05 was considered to be significant. Missing data or loss to follow-up, if any, will be addressed in results section.

## 3 Results

### 3.1 Patient demographics

Figure 1 shows a flow chart of the process used for selecting the study participants. In total, 281 patients were initially diagnosed with sepsis by the ED physician, but 106 were excluded as they had no available samples (as detailed in section 2.2.). Therefore, 175 patients were initially eligible for biomarker measurements. After a retrospective re-evaluation by an independent infectious diseases specialist, 15 patients were excluded as they did not meet the diagnostic criteria for sepsis. Of the final 160 study participants, 78 had sepsis and 82 had septic shock.

The baseline characteristics of the study population are summarized in Table 1. Among the 160 patients, 97 were survivors and 63 were non-survivors (28-day mortality rate: 39.4%). Overall, the most common site of infection was the respiratory system (n = 102; 63.8%), followed by the genitourinary (n = 48; 30.0%) and gastrointestinal systems (n = 13; 8.1%).

**Table 1:**
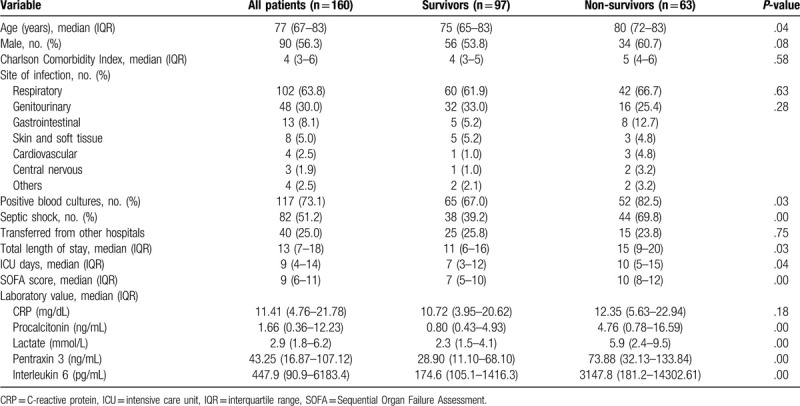
Baseline characteristics of the study population.

There were no participants with missing data for each variable of interest. There were no participants with loss to follow-up. None of continuous variables were categorized in the present study.

### 3.2 Prognostic value of each biomarker and Sequential Organ Failure Assessment score

**Figure 2:**
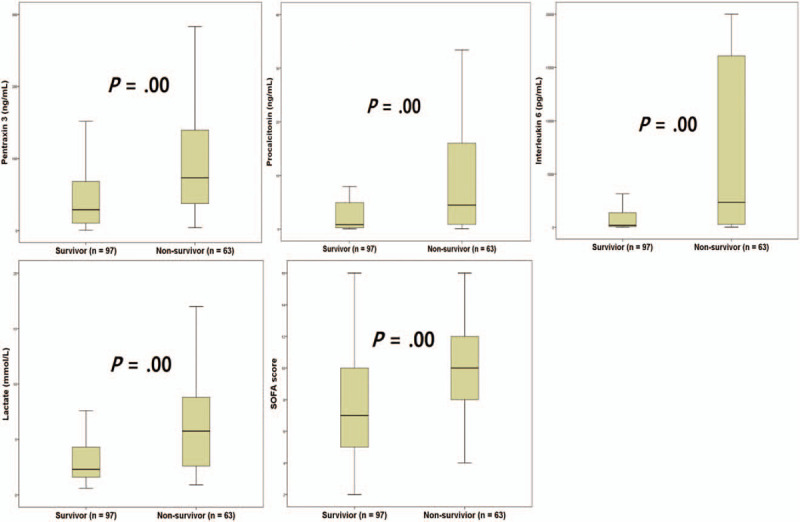
Procalcitonin, lactate, pentraxin 3, interleukin 6, and Sequential Organ Failure Assessment score in survivors and non-survivors (on the 28th day).

**Figure 3:**
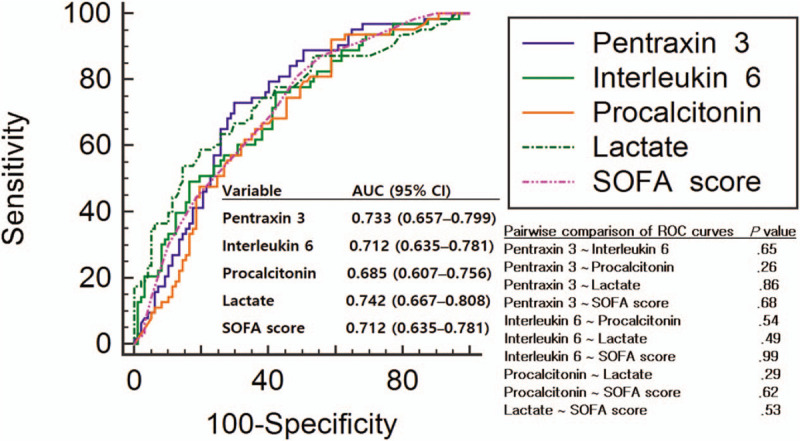
Receiver operating characteristic curves of each biomarker and Sequential Organ Failure Assessment score for predicting 28-day mortality.

The levels of PCT, lactate, PTX3, and IL6 are presented in Table 1. In the comparison between non-survivors and survivors, the SOFA scores and concentrations of the 4 biomarkers were higher in the non-survivors (all *P* = .00) (Fig. 2). The optimal cutoff values for 28-day mortality were as follows: lactate, 5.45 mmol/L; PCT, 0.47 ng/mL; IL6, 269.47 pg/mL; PTX3, 26.90 ng/mL; and SOFA score, 8 (Table 2). The sensitivity and specificity of each cutoff value are also shown in Table 2. Aside from PCT (AUC: 0.685), all the other variables showed a fair performance (AUC: 0.712–0.742) in predicting 28-day mortality (Table 2; Fig. 3). However, there were no differences between the AUCs (pairwise comparison of ROC curves) (Fig. 3).

**Table 2:**
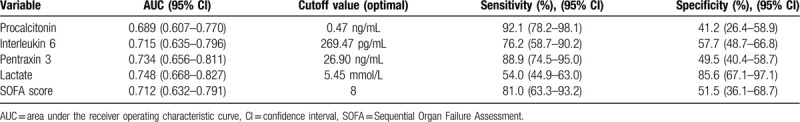
Optimal cutoff values of four biomarkers and the Sequential Organ Failure Assessment score for predicting 28-day mortality in patients with sepsis.

### 3.3 Combination of Sequential Organ Failure Assessment score and the 4 biomarkers

#### 3.3.1 Using the frequency sum of values above the cutoff

**Figure 4:**
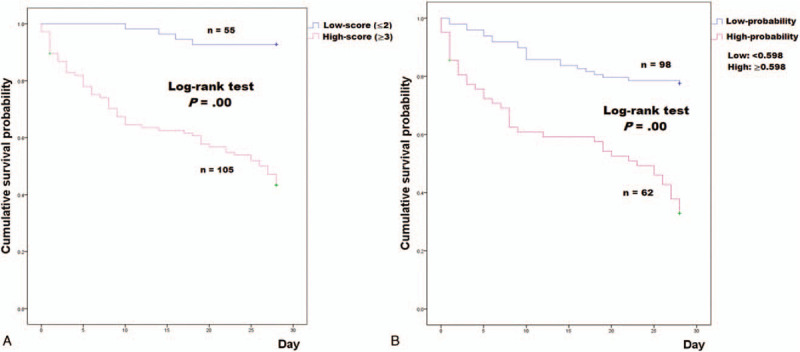
Kaplan–Meier curve according to the (A) frequency sum of values above the cutoff and (B) multivariate logistic regression model.

**Figure 5:**
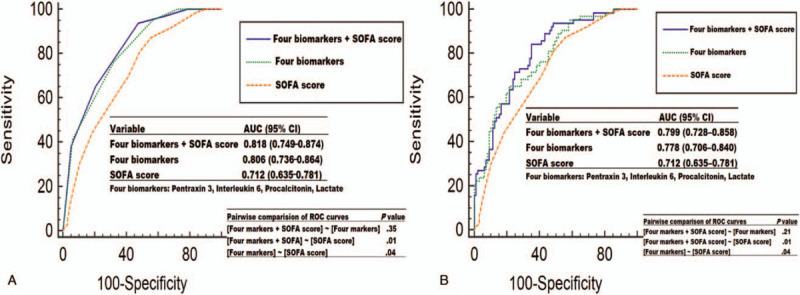
Receiver operating characteristic curves of each variable for predicting 28-day mortality. (A) Assessed using the frequency sum of values above the cutoff. (B) Assessed using the multivariate logistic regression model (probability of 28-day mortality). AUC = area under the receiver operating characteristic curve, CI = confidence interval, SOFA = Sequential Organ Failure Assessment.

The combination of each biomarker and SOFA score using the frequency sum of values above the cutoff is presented in Table 3. The 28-day mortality rate was different among the 5 groups, with a stepwise increase: 0% in group 0, 12.5% in group 1, 11.1% in group 2, 40.9% in group 3, 53.1% in group 4, and 82.8% in group 5. Figure 4A depicts the Kaplan–Meier curve of 28-day mortality stratified by the frequency sum of values above the cutoff (≥3 vs ≤2). The high-score group (≥3) showed a significantly higher 28-day mortality rate than the low-score group (≤2) (56.2% vs 7.3%, respectively; log-rank test, *P* = .00). In the ROC curve analysis, the combination of the 4 biomarkers was better than the SOFA score (AUC: 0.806 (95% CI: 0.736–0.864) vs 0.712 (95% CI: 0.635–0.781); *P* = .01) (Fig. 5A) in predicting 28-day mortality. The addition of the SOFA score to the 4 biomarkers did not result in a better performance (AUC: 0.818 [95% CI: 0.749–0.874]) than that of the 4 biomarkers combined (*P* = .35) (Fig. 5A).

**Table 3:**
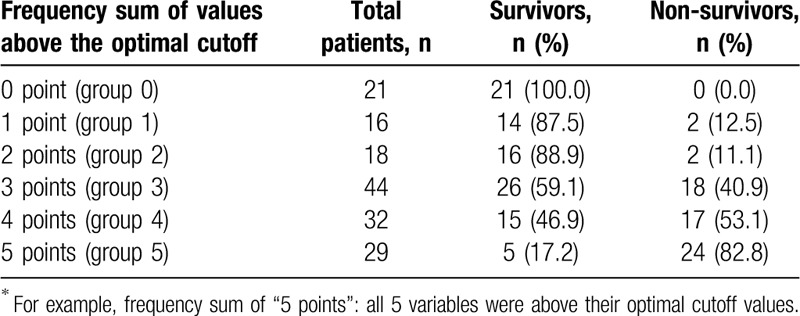
The 28-day mortality rates according to the frequency sum of values above the optimal cutoff, using four biomarkers and the Sequential Organ Failure Assessment score^∗^.

#### 3.3.2 Using the multivariate logistic regression model

A multivariate logistic regression model was constructed to predict the 28-day mortality rate using the 4 biomarkers combined. In the final step of the regression analysis, PTX3, lactate, PCT, and IL6 remained statistically significant. Using the regression equation, the log of probability was converted to the probability of 28-day mortality as follows: 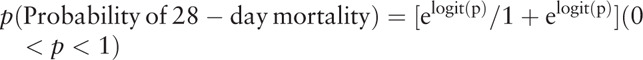


The Hosmer–Lemeshow chi-square value was 9.313 (*P* = .317 > .005, 8 degrees of freedom), indicating that the model had a good calibration. The optimal cutoff value for the probability of 28-day mortality was .598, with a sensitivity of 65.1% and specificity of 78.4%. Fig Figure 4B depicts the Kaplan–Meier curve of 28-day mortality stratified by the cutoff values for the probability of 28-day mortality. The 28-day mortality rates were 22.4% and 66.1% in the low- and high-probability groups, respectively. The high-probability group (≥.598) showed a significantly higher 28-day mortality rate than the low-probability group (<.598) (log-rank test, *P* = .00). In the ROC curve analysis, the biomarker combination was better than the SOFA score (AUC: 0.778 (95% CI: 0.706–0.840) vs. 0.712 (95% CI: 0.635–0.781); *P* = .04) (Fig. 5B) in predicting 28-day mortality. The addition of the SOFA score to the four biomarkers did not result in a better performance (AUC: 0.799 (95% CI: 0.728–0.858)) than that of the 4 biomarkers combined (*P* = .21) (Fig. 5B).

### 3.4 Cox proportional hazards models of risk factors for 28-day mortality

Unadjusted and adjusted HRs of the biomarkers and SOFA score derived from the Cox proportional hazards model are presented in Table 4. A multivariate Cox proportional model adjusted for age, positive blood culture, CRP, and septic shock revealed that all the variables (PTX3, IL6, PCT, lactate, and SOFA score) were the risk factors for 28-day mortality.

**Table 4:**
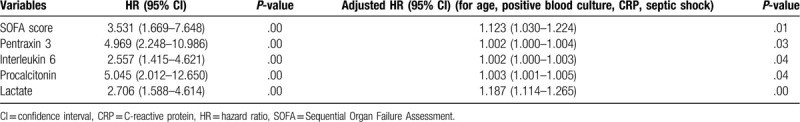
Univariate and multivariate Cox proportional hazards models of risk factors for 28-day mortality.

### 3.5 Subgroup analysis and sensitivity analysis

Subgroup analysis according to the disease severity (sepsis vs septic shock) found higher AUC in sepsis group than that in septic shock group (Table 5). Two sensitivity analyses were conducted to test the model assumptions. First, when severe sepsis and septic shock were defined by using the previous Sepsis-2 definitions (5 sepsis patients excluded from the sensitivity analysis), AUCs (95% CI) of biomarker combination plus SOFA score were 0.802 (0.729–0.853) in frequency sum of values above the cutoff model and 0.788 (0.713–0.842) in multivariate logistic regression model, respectively. Second, when we excluded patients who were transferred from other institutions, AUCs (95% CI) of biomarker combination plus SOFA score were 0.798 (0.725–0.851) in frequency sum of values above the cutoff model and 0.783 (0.707–0.838) in multivariate logistic regression model, respectively. Both of the sensitivity analyses returned results similar to those of the main analysis. Additionally, Table 6 shows the levels of each biomarker according to the sites of infection. There were no significant differences in the levels of the overall biomarkers between respiratory and genitourinary infection. Both respiratory and genitourinary infection had higher mortality rate than gastrointestinal infection in sepsis patients.

**Table 5:**
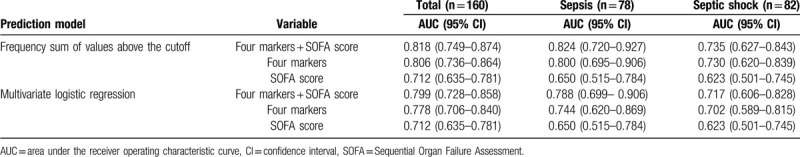
Predictive value of the 2 different models stratified by the disease severity (sepsis vs septic shock).

**Table 6:**
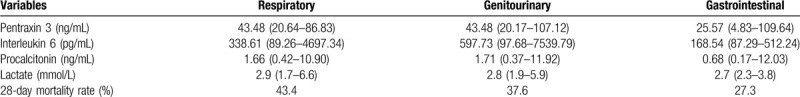
The levels of each biomarker according to the sites of infection.

## 4 Discussion

Our present study had some strengths. First, we investigated the prognostic value of individual biomarkers (PTX3, PCT, IL6, and lactate) or their combination in patients diagnosed with sepsis or septic shock according to the latest Sepsis-3 definitions. Second, this prospective observational study validated the performance of the combined biomarker approach in predicting 28-day mortality, using 2 different methods: the frequency sum of values above the cutoff, and the multivariate logistic regression model. Both analyses showed that the combined biomarker approach predicted 28-day mortality with good performance (AUC: 0.806 and 0.778, respectively) and was superior to the SOFA score in this regard (AUC: 0.712). Furthermore, the biomarker combination was a valuable supplement to the SOFA score for the prediction of 28-day mortality among all patients.

We had previously investigated the diagnostic value of PTX3, IL6, and PCT individually in patients with sepsis or septic shock,^[17]^ where the results showed that each biomarker had good value in discriminating these patients from control patients who had no infections. This present study included the patients (n = 97) who had been enrolled in our previous study and newly recruited patients (n = 63), to validate the performance of the combined biomarker approach in predicting 28-day mortality.

Lactate is one of the most widely used and valuable biomarkers for predicting mortality in patients with sepsis. According to a published review article, the lactate level has been used as a marker of disease severity and a predictor of mortality in patients with sepsis.^[18]^ However, 2 other studies reported that the use of lactate alone as the biomarker showed only fair (not good) predictive value for 28-day mortality (AUC: ≤0.70; poorer than that observed in our study).^[8,19]^ Likewise, PCT, PTX3, and IL6 had a moderate prognostic value in sepsis,^[9,10,13,19–21]^ but the value of each single marker was limited.

Because of the limitation of the single marker approach, several studies have investigated the prognostic value of biomarker combinations in predicting mortality.^[16,19,21,22]^ In accordance with the present study, these studies showed that the combined biomarker approach had a better prognostic value than the single marker approach. Our study is novel with respect to the combination of lactate, PCT, PTX3, and IL6 in predicting mortality, using 2 different methods: the frequency sum of values above the cutoff, and the multivariate logistic regression model.

A recent cohort study had shown that an increase in the SOFA score of 2 or more had greater prognostic accuracy for in-hospital mortality than the systemic inflammatory response syndrome (SIRS) criteria or qSOFA score.^[23]^ However, 2 other recent studies had demonstrated that the combined biomarker approach had a better prognostic value than the SOFA score in predicting 30-day mortality.^[16,21]^ In accordance with our results, the 2 studies commonly showed that the combination of multiple biomarkers and the SOFA score performed better than the multiple marker approach or SOFA score alone.^[16,21]^

The levels of each biomarker according to the sites of infection were shown in Table 6. Respiratory and genitourinary infection were the most common causes of sepsis in the present study. There were no significant differences in the levels of the 4 biomarkers between 2 infection sites. Both respiratory and genitourinary infections had higher mortality rate than gastrointestinal infection in sepsis patients. Further studies with the larger study population are needed to investigate the association between source of infection and levels of biomarkers.

This study had some limitations. First, because the study was conducted in a single tertiary teaching hospital, it remains uncertain whether the results are generalizable to external populations. Second, because numerous distinct biomarkers have been proposed as potential biomarkers for predicting mortality in patients with sepsis, the real challenge is the optimal selection and validation of useful biomarkers from the huge pool of potential candidates.^[6,24]^ Even though we have identified the prognostic value of the combination of the 4 biomarkers selected, there could be more valuable combinations using other novel biomarkers. Third, the present study included patients with sepsis or septic shock who met the positive qSOFA criteria upon arrival to the ED, which might have resulted in a selection bias.

## 5 Conclusions

The combined biomarker approach using PTX3, PCT, IL6, and lactate showed good performance in predicting 28-day all-cause mortality among the patients diagnosed with sepsis or septic shock as defined by Sepsis-3. The prognostic value of this approach was superior to that of the SOFA score and could therefore be a supplement to the SOFA score for predicting mortality. Future multicenter studies are needed to further investigate prognostic value of biomarker combination in accordance with the Sepsis-3 definitions.

## Acknowledgments

The authors are grateful to researchers Hye-yoon Jung and Min-sook Jung for their contributions to the project.

## Author contributions

**Conceptualization:** Juhyun Song, Sungwoo Moon, Dae Won Park.

**Data curation:** Juhyun Song, Joo Yeong Kim, Jae Hyung Cha

**Formal analysis:** Juhyun Song, Jae Hyung Cha

**Investigation:** Juhyun Song, Sungwoo Moon, Jonghak Park.

**Methodology:** Juhyun Song, Dae Won Park.

**Project administration:** Sungwoo Moon, Dae Won Park.

**Resources:** Juhyun Song, Sungwoo Moon, Dae Won Park, Han-jin Cho.

**Software:** Juhyun Song, Jae Hyung Cha

**Supervision:** Sungwoo Moon, Dae Won Park.

**Visualization:** Juhyun Song.

**Writing – original draft:** Juhyun Song, Sungwoo Moon.

**Writing – review & editing:** Dae Won Park, Han-jin Cho.
